# Combating Loneliness With Nostalgia: Nostalgic Feelings Attenuate Negative Thoughts and Motivations Associated With Loneliness

**DOI:** 10.3389/fpsyg.2020.01219

**Published:** 2020-06-23

**Authors:** Andrew A. Abeyta, Clay Routledge, Samuel Kaslon

**Affiliations:** ^1^Department of Psychology, Rutgers, The State University of New Jersey, Camden, NJ, United States; ^2^Department of Psychology, North Dakota State University, Fargo, ND, United States

**Keywords:** loneliness, nostalgia, social goals, social approach, social-confidence

## Abstract

Loneliness is difficult to overcome, in part because it is associated with negative social cognitions and social motivations. We argue that nostalgia, a positive emotional experience that involves reflecting on cherished memories, is a psychological resource that regulates these maladaptive intrapsychic tendencies associated with loneliness. We tested this hypothesis across 4 studies. Study 1 examined whether nostalgia mitigates the inverse relation between loneliness and social confidence. Studies 2, 3, and 4 examined nostalgia’s potential to mitigate the inverse relation between loneliness and approach-oriented social goals and intentions. The results provided support that nostalgia mitigates reduced social confidence and low approach-oriented social goals/intentions associated with loneliness. The associations between loneliness and reduced social confidence, and loneliness and less approach-oriented social goals/intentions, respectively, were found to be weaker as a function of nostalgia. This weakening appeared to be due to nostalgia’s positive effect on social confidence and approach-oriented social goals/intentions, respectively, particularly at high levels of loneliness.

## Introduction

In an age in which technology has made connecting with others easier than ever before, people are surprisingly feeling increasingly lonely. For example, a report published by the Jo Cox Commision on Loneliness (2017) found that more than 9 million British adults report chronic feelings of loneliness. Moreover, a large-scale study of loneliness using data from older adults from 2008 and 2012 in the United States found that upward of 40% of adults over 60 feel lonely at least some of the time (Gerst-Emerson and Jayawardhana, 2015). A wealth of research has demonstrated that chronic loneliness is a serious health risk. Specifically, chronic loneliness is linked to deficits in psychological (Cacioppo et al., 2006) and physical health (for a review, see Cacioppo and Cacioppo, 2014).

Loneliness comes from feeling that the quantity and/or quality of one’s interpersonal relationships are unfulfilling or less than desired, which then leads to the persistent feeling that an individual is alone (Cacioppo et al., 2015). Thus, people suffering from chronic loneliness would want to establish positive social connections (Gardner et al., 2005). However, research indicates that loneliness is associated with low confidence about one’s ability to succeed in the interpersonal domain (Solano and Koester, 1989; Cacioppo et al., 2006) and a maladaptive interpersonal motivation/goal orientation of being less inclined to pursue approach-oriented social goals (Park and Baumeister, 2015). Ultimately, these negative intrapsychic tendencies make successful social connections unlikely. Individuals who think negatively about their interpersonal competencies and who are reluctant to pursue approach-oriented interpersonal goals, such as interpersonal growth, struggle to maintain satisfying relationships (e.g., Gable, 2006; Vanhalst et al., 2015). Moreover, intervention research indicates that regulating negative thoughts and goal orientations is important for overcoming chronic loneliness. Specifically, interventions designed primarily to address the underlying negative intrapsychic tendencies associated with loneliness are more effective than interventions primarily focused on increasing opportunities for social contact, interventions focused on increasing social support, and interventions to improve social skills (Masi et al., 2011; Cacioppo et al., 2015). Therefore, it is important to identify psychological resources that are associated with or may encourage more adaptive social confidence and a more adaptive approach-oriented goal pattern among lonely individuals. We propose that nostalgia is a psychological resource that regulates loneliness by lessening the relation between loneliness and social confidence, and the relation between loneliness and reduced approach-oriented social goals.

## Nostalgia

Even though nostalgia was once considered a disease or mental illness (for a reviews, see Batcho, 2013), contemporary treatments of this construct are in agreement that nostalgia is an emotionally complex but mostly positive experience. Laypersons, for example, consider nostalgia to be a mostly positive experience with elements of loss, as well as a revisiting of fond and personally significant memories that are primarily focused on childhood and/or social relationships (Hepper et al., 2012, 2014). Research exploring the content of nostalgic memories is consistent with laypersons’ definitions of nostalgia. Specifically, this evidence indicates that nostalgia is primarily a positive emotional experience (Wildschut et al., 2006; Abeyta et al., 2015b) that is self-focused (i.e., the self plays the role of protagonist) but also social in nature, referencing feelings of love/belonging and featuring meaningful social relationships and events (Wildschut et al., 2006; Abeyta et al., 2015b).

Although pleasant sensory inputs, such as familiar scents (Reid et al., 2015) or music (e.g., Routledge et al., 2011; Abeyta et al., 2015a), have been found to induce nostalgia, people most often turn to nostalgia in distressing or threatening contexts. For example, research indicates that negative mood inductions (Wildschut et al., 2006), threats to the self (e.g., Vess et al., 2012), and challenges to a sense of meaning in life (Routledge et al., 2011) bring on line nostalgia. Most relevant to the current paper, loneliness has been found to be a potent trigger of nostalgia (Wildschut et al., 2006; Zhou et al., 2008).

People turn to nostalgia in response to distress because a wealth of research indicates that nostalgic reverie has a number of psychological benefits (e.g., Routledge et al., 2013). First, nostalgia increases positive, but not negative affect (e.g., Wildschut et al., 2006). Second, nostalgia promotes positive self-views (Vess et al., 2012), promotes authenticity (Baldwin et al., 2015), fosters self-continuity (e.g., Sedikides et al., 2015), and bolsters self-esteem (e.g., Wildschut et al., 2006; Cheung et al., 2013). Third, nostalgia has existential benefits. Nostalgia bolsters a sense of meaning in life (Routledge et al., 2011) and buffers a variety of existential threats (e.g., Routledge et al., 2008, 2014). Fourth and most relevant to the current work, nostalgia bolsters a sense of social connectedness (i.e., a sense of acceptance, belongingness, and support, e.g., Wildschut et al., 2006, 2010; Juhl et al., 2012), increases feelings of social competence (Wildschut et al., 2006; Abeyta et al., 2015a), and energizes interpersonal goals of connecting with others and deepening relationships (Abeyta et al., 2015a).

## The Social Regulatory Benefits of Nostalgia

### Nostalgia Reduces Loneliness via Social Support

Being that nostalgia is an experience that people naturally turn to when feeling lonely and that nostalgia fosters social connectedness, it should regulate loneliness by fostering feelings of social support. Indeed, Zhou et al. (2008) found that loneliness was associated with greater nostalgia proneness (i.e., the propensity to engage in nostalgic reflection), and nostalgia proneness was, in turn, associated with greater feelings of social support. Critically, nostalgia proneness suppressed the relation between loneliness and social support; when statistically controlling for nostalgia proneness, the association between loneliness and social support became more strongly negative. In the same paper, Zhou et al. (2008) found that manipulated loneliness increased nostalgic feelings and decreased perceptions of social support. Zhou and colleagues conducted a mediation analysis by first testing the effects of the loneliness manipulation on nostalgic feelings and social support separately and then examining the effect of the loneliness manipulation on social support while controlling for the relationship between nostalgic feelings and social support. The results were that the loneliness manipulation increased nostalgic feelings but decreased social support. Moreover, when statistically controlling for the positive relationship between nostalgic feelings and social support, the effect of the loneliness manipulation on social support was more strongly negative. Thus, Zhou and colleagues provided evidence that the tendency to recruit nostalgia in response to loneliness suppressed the effect of loneliness on reduced social support.

### Nostalgia Regulates Loneliness via Maladaptive Intrapsychic Tendencies

In addition to a lack of social support, loneliness has been linked to negative intrapsychic tendencies that undermine people’s ability to successfully connect with others. For example, loneliness is associated with negative thoughts about social interactions and with a reduced sense of social-confidence (Solano and Koester, 1989; Cacioppo et al., 2006). Negative attitudes about social success and a lack of social confidence have been linked to difficulty communicating with other people (Solano and Koester, 1989) and a decreased desire to pursue social contact (Vanhalst et al., 2015), both of which can contribute to the loss of social bonds (Duck et al., 1994). Additionally, loneliness is inversely associated with an approach or promotion-oriented goal focus (Park and Baumeister, 2015). People who are less inclined to pursue approach-oriented social goals such as intimacy experience negative interpersonal outcomes (Elliot et al., 2006; Gable, 2006).

In sum, negative intrapsychic tendencies of low social confidence and reduced social approach goal focus associated with loneliness set lonely people up for unsatisfying interpersonal interactions, potentially exacerbating loneliness. There is reason to believe that nostalgia regulates these negative social cognitions and goal orientations. Nostalgic reminiscence typically involves people revisiting their most cherished social memories (Wildschut et al., 2006; Abeyta et al., 2015a, b), an experience that has been found to strengthen perceptions of confidence in the interpersonal domain (Abeyta et al., 2015a). Research suggests that nostalgia may also be involved in regulating the tendency for lonely people to be less motivated to pursue approach-oriented social goals. Nostalgia is theorized as an active coping resource that regulates distress and instigates fundamental energies aimed at realizing positive end states (e.g., Stephan et al., 2014). Indeed, research indicates that distressing situations trigger nostalgia and nostalgia promotes positive emotions and views of the self (Wildschut et al., 2006). Building on this research, Stephan et al. (2014) provided evidence that nostalgia generally regulates avoidance-related motivation and increases approach-related motivation (Stephan et al., 2014; Tullett et al., 2015). Moreover, a recent study found that induced nostalgia reduced the amplitude of the event-related negativity, a neural marker of defensive motivation (Bocincova et al., 2019). According to the hierarchical model of approach-avoidance motivation (Elliot and Church, 1997), motivation, defined as disposition or triggered action tendencies, leads to the adoption of specific goals. Building on the hierarchical model of approach-avoidance motivation (Elliot and Church, 1997), research that nostalgia instigates approach motivation (Stephan et al., 2014), and research that nostalgia is prototypically focused on social relationships (e.g., Hepper et al., 2014; Abeyta et al., 2015b). Abeyta et al. (2015b) proposed that nostalgia promotes approach-oriented social goals such as growth, intimacy, and interpersonal repair. Indeed, this research found that engaging in nostalgic reverie energized approach-oriented goals of establishing, deepening, and repairing social bonds (Abeyta et al., 2015a).

## The Present Research

The purpose of the current research was to test the predictions that nostalgic feelings regulate negative thoughts about ones’ ability to succeed in interpersonal relationships and weak goals/intentions for establishing deep bonds that are associated with loneliness. There are two possible models as to how nostalgia may operate to regulate loneliness via intrapsychic tendencies; nostalgia may mediate the relation between loneliness and negative intrapsychic tendencies or moderate the relation. The mediation model supposes that nostalgia is a response to loneliness, because nostalgia tends to involve longing for relationships from the past. Research supporting the mediation model has demonstrated that people identify loneliness as a common trigger for nostalgic reverie (Wildschut et al., 2006), that manipulated loneliness increases nostalgic feelings (Wildschut et al., 2006; Zhou et al., 2008), and that individuals high in trait loneliness report engaging in nostalgia more frequently (Zhou et al., 2008). As a response to loneliness, the mediation model proposes that nostalgia regulates loneliness. As previously mentioned, research showing that trait nostalgia suppresses the inverse relation between trait loneliness and perceptions of social support supports the mediation model (Zhou et al., 2008).

The moderation model also proposes that nostalgia functions to regulate loneliness but does not treat nostalgia as an inevitable response to loneliness. This model has been used to explore the conditions under which people turn to nostalgia in response to loneliness or lack of social belonging. Research investigating when people turn to nostalgia in response to loneliness has shown, for example, that the association between trait loneliness and nostalgia is stronger among individuals high in psychological resilience (Zhou et al., 2008). Such findings may help explain why the correlation between loneliness and nostalgia is often small (Zhou et al., 2008). For the present research, we based our hypothesis on the moderation model because our intention was to investigate how the association between trait loneliness and negative intrapsychic tendencies change when people are experiencing state nostalgic feelings, compared to when they are not. However, the correlational nature of Studies 1–3 allowed us to also consider the mediation model prior to the experimental approach taken in Study 4.

In Studies 1–3, we explored this non-experimentally. Whereas past research testing the mediation model measured nostalgia and loneliness as traits (Zhou et al., 2008), we endeavored to measure loneliness as a trait and nostalgia as a state. We accomplished this by instructing participants to think about their general experiences when completing the loneliness inventory but consult their feelings in the moment when completing the nostalgia inventory. In addition to trait loneliness and state nostalgia, we measured state feelings of social confidence (Study 1) and approach-oriented social goals/intentions (Studies 2 and 3). Consistent with past research on the association between loneliness and negative intrapsychic tendencies (e.g., Cacioppo et al., 2006; Park and Baumeister, 2015), we hypothesized that trait loneliness would be associated with lower social confidence in Study 1 (Hypothesis 1a) and that trait loneliness would be associated with lower less approach-oriented social goals/intentions (Hypothesis 1b). Consistent with past research that nostalgia promotes social confidence and increases commitment to approach-oriented social goals (Abeyta et al., 2015a), we hypothesized that nostalgic feelings would be positively associated with social confidence in Study 1 (Hypothesis 2a) and that nostalgic feelings would be positively associated approach-oriented goals in Studies 2 and 3 (Hypothesis 2b).

While there is ample evidence that trait loneliness is associated with nostalgia proneness (e.g., Wildschut et al., 2006; Zhou et al., 2008), we did not anticipate a strong correlation between trait loneliness and state nostalgic feelings. Lonely people may be more prone to engage in nostalgic reverie because they tend to experience situations that trigger feelings of loneliness or social exclusion more frequently (Hanley-Dunn et al., 1985) and triggered feelings of loneliness increase nostalgic feelings (e.g., Wildschut et al., 2006). However, it is uncertain whether lonely people experience nostalgia when environmental triggers of loneliness are lacking. Measuring trait loneliness and state nostalgia allowed us to test this question.

The lack of strong correlation would enable us to test whether the relationship between trait loneliness and negative intrapsychic tendencies is impacted when people naturally experience nostalgia. In Study 1, we hypothesized that the relation between loneliness and lower social confidence would be weaker as a function of increased nostalgic feelings, because stronger feelings of nostalgia would correspond with greater social confidence, particularly at higher levels of loneliness (Hypothesis 3a). In Studies 2 and 3, we hypothesized that the relation between loneliness and reduced approach-oriented social goals/intentions would be weaker as a function of increased nostalgic feelings, and approach-oriented social goals/intentions, particularly at higher levels of loneliness (Hypothesis 3b).

In Study 4, we adopted an experimental approach to investigate the potential for nostalgia to regulate the negative intrapsychic tendencies associated with loneliness. We manipulated loneliness instead of measuring it, and rather than rely on naturally occurring feelings of nostalgia we induced nostalgia. Additionally, we assessed approach-oriented social intentions. Once again, we expected nostalgia to moderate the impact of loneliness on reduced approach-oriented goal focus, by increasing approach-oriented social intentions, particularly at high levels of loneliness (Hypothesis 3c).

### Sample Size Determination

We planned to test for the main effects of loneliness and nostalgia, as well as their interaction, using hierarchical linear regressions in Studies 1, 2, and 3, and a 2 × 2 between-subjects analysis of variance (ANOVA) in Study 4. We calculated the minimal sample size for all studies using G^∗^Power version 3.1.9.2 (Faul et al., 2007). Based on our design for Studies 1, 2, and 3, the small to medium effect size was *f*^2^ = 0.08, power was 0.80, alpha was set at 0.05, and the required minimum sample size was 139, but in all cases, larger samples were secured. Based on our design for Study 4, the small to medium effect size was η*_*p*_*^2^ = 0.03, power was 0.80, alpha was set at 0.05, and the minimum sample size was 156, and therefore, we endeavored to recruit at least 200 participants. We ran Study 4 on Amazon Mechanical Turk (AMT) and manipulated loneliness with a false-feedback paradigm. Because recent research suggests that AMT participants are experienced with deception in psychological research and that this familiarity can undermine the validity of experimental paradigms (for a review see, Hauser et al., 2019), we decided to oversample. As a rough rule of thumb, we planned on recruiting double the desired sample size.

## Study 1

The purpose of Study 1 was to investigate nostalgia’s potential to regulate the tendency for chronically lonely individuals to hold negative attitudes about their ability to attain/maintain interpersonal relationships (e.g., Cacioppo et al., 2006). Specifically, we tested whether nostalgic feelings moderate the inverse relation between loneliness and social confidence. We accomplished this aim by assessing trait loneliness, state nostalgia, and feelings of social confidence. We hypothesized that loneliness would be associated with reduced social confidence. However, we expected that this association would be weaker when people report feeling more nostalgic, because stronger feelings of nostalgia would be associated with social confidence, particularly at higher levels of loneliness.

### Materials and Methods

#### Participants

Participants consisted of 208 undergraduate students from a mid-Atlantic university (147 female participants) who took part in the study for course credit. Participants’ ages ranged from 18 to 59 years (*M* = 20.11, *SD* = 4.13).

#### Procedure and Materials

Participants completed an online questionnaire consisting of a loneliness questionnaire, a nostalgia inventory, and a social confidence scale.

##### Loneliness

Participants completed the 10-item UCLA loneliness questionnaire (Russell, 1996). Specifically, participants indicated the extent to which they feel deprived of companionship, feel isolated, and generally lack support using a 4-point response scale (e.g., “How often do you feel like people are around you but not with you?”; 1 = *never*, 4 = *always;*α = 0.88; *M* = 2.38, *SD* = 0.54).

##### Nostalgia

A state version of the Nostalgia Inventory (Batcho, 1995) was used as a measure of state nostalgia, where participants were specifically instructed to respond to the Nostalgia Inventory based on how they were feeling in the moment. Participants are presented with a dictionary definition of nostalgia (i.e., “*According to the Oxford Dictionary, ‘nostalgia’ is defined as ‘a sentimental longing for the past’*”) and self-report how nostalgic they are currently feeling about 20 different aspects (e.g., “family,” “places”) of their past (1 = *not at all nostalgic*, 7 = *very nostalgic*; α = 0.86; *M* = 4.78, *SD* = 0.97).

##### Social confidence

A 6-item scale was used to assess confidence in establishing and maintaining relationships (Abeyta et al., 2015a). Specifically, participants read the following stem, “Rate your confidence in your ability to…” and then responded to the following six items: “…establish successful social relationships,” “…maintain social relationships,” “…resolve conflicts in relationships,” “…communicate effectively in social relationships,” “…open up to others in social relationships,” and “…approach people I don’t know and strike up a conversation” (1 = *cannot do at all*, 10 = *highly certain can do*; α = 0.89; *M* = 7.42, *SD* = 1.77).

### Results

First, we conducted correlations between the measured variables. Loneliness was significantly and negatively correlated with social confidence. State nostalgia was significantly and positively associated with social confidence. Inconsistent with the mediation model, loneliness was negatively and weakly correlated with state nostalgia. These correlations is found in Table 1.

**TABLE 1:** Bivariate correlations for Studies 1, 2, and 3.

	Study 1		Study 2			Study 3			
Factor	Nostalgia	Social efficacy	Nostalgia	Social approach	Proactive social intentions	Nostalgia	Social approach	Participation in social studies	Participation in non-social studies
Nostalgia	–	0.20*	–	0.33**	0.27**	–	0.27**	0.08	0.15*
Loneliness	-0.11	−0.48**	−0.15*	−0.56**	−0.43**	0.01	−0.27**	–0.06	0.12

*p < 0.05, **p < 0.001.

Next, we conducted hierarchical linear regression analyses regressing social confidence on state nostalgia (centered at the mean) and loneliness (centered at the mean) in the first step. In the second step, we added the state nostalgia × loneliness interaction. This overall equation was significantly predictive of social confidence, *R*^2^ = 0.27, *F*(3, 204) = 24.46, *p* < 0.001.

This analysis revealed a significant main effect of loneliness on social efficacy, such that higher loneliness was associated with lower levels of social efficacy, *b* = −1.52, *SE* = 0.20, *t* = −7.55, *p* < 0.001, *sr*^2^ = 0.21, 95% CI [−1.91, −1.12]. There was also a significant main effect of state nostalgia, such that greater feelings of nostalgia were associated with more social efficacy, *b* = 0.26, *SE* = 0.11, *t* = 2.36, *p* = 0.019, *sr*^2^ = 0.02, 95% CI [0.04, 0.48]. These main effects were qualified by a significant loneliness × state nostalgia interaction, *b* = 0.44, *SE* = 0.20, *t* = 2.20, *p* = 0.03, *sr*^2^ = 0.02, 95% CI [0.05, 0.84].

**FIGURE 1:**
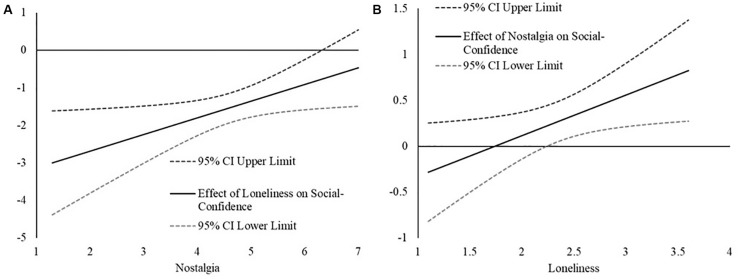
**(A)** The plot represents the estimated relation between loneliness and social confidence (*y*-axis) across the range of nostalgic feelings (*x*-axis) in Study 1. The relations are considered statistically significant if the confidence intervals for the effect (CI) do not contain 0. **(B)** The plot represents the estimated relation between nostalgic feelings and social confidence (*y*-axis) across the range of loneliness scores (*x*-axis) in Study I. The relations are considered statistically significant if the confidence intervals for the effect (Cl) do not contain 0.

We probed the interaction with the Johnson and Neyman (1936) technique. The advantage of this technique is that it estimates the effect of loneliness on social confidence along the full range of nostalgia scores (Hayes and Matthes, 2009), allowing us to see how the association weakens or strengthens as a function of state nostalgia. First, we examined how the relation between loneliness and social confidence changed as a function of nostalgia. In Figure 1A, we have plotted the estimated effect of loneliness on social confidence across the range of state nostalgia scores and, consistent with the hypothesis, the strength of the loneliness and social confidence relation weakens (becomes less strongly negative) as a function of greater state nostalgia, becoming non-significant at very high levels of state nostalgia (greater than 6.32, between 1 and 2 SD above the mean).

For the sake of being thorough, we also examined how the relation between nostalgic feelings and social confidence as a function of loneliness. The Johnson and Neyman (1936) technique revealed that the relation between nostalgia and social confidence became more strongly positive as a function of greater loneliness, becoming statistically significant at moderate levels of loneliness (2.35, slightly below the mean; see Figure 1B).

Consistent with past research (e.g., Cacioppo et al., 2006), trait loneliness was associated with deficits in social confidence. Also consistent with past research (e.g., Abeyta et al., 2015a), nostalgic feelings were associated with greater social confidence. Critically, nostalgia moderated the relation between loneliness and negative social cognitive tendencies, because of its positive association with social-confidence. The association between loneliness and social-efficacy was found to be strongest at very low levels of nostalgia but to be weaker at higher levels of nostalgia. Moreover, this weakening appears to be because of nostalgia’s positive association with social confidence. As can be seen in Figure 1A, social confidence is relatively high at low levels of loneliness and does not vary as a function of nostalgia. However, social confidence does vary as a function of state nostalgia at higher levels of loneliness, such that lonely individuals who report stronger nostalgic feelings report less of a deficit in social confidence compared lonely individuals who report weaker nostalgic feelings.

## Study 2

An important question is whether nostalgia’s impact goes beyond feelings of confidence to social goals about connecting with others. Therefore, the purpose of Study 2 was to focus on social goals/intentions. Past research has established that loneliness is associated with reduced approach-oriented goals (Park and Baumeister, 2015). We tested whether nostalgic feelings moderate this inverse relation by assessing trait loneliness, state nostalgia, and approach-oriented social goals/intentions. We hypothesized that loneliness would be associated with reduced commitment to approach-oriented social goals. However, we expected nostalgia to moderate this relation, such that the inverse relation would be weaker as a function of nostalgia, because nostalgic feelings are positively associated with approach-oriented social goals, particularly at higher levels of loneliness.

### Materials and Methods

#### Participants

Participants consisted of 200 Amazon Mechanical Turk (AMT) workers residing in the United States (98 female participants). AMT is a valid and reliable source for psychological research (Paolacci et al., 2010; Buhrmester et al., 2011; Shapiro et al., 2013). AMT samples are comparable to traditional samples (e.g., college, community, and clinical samples) on demographic measures (Paolacci et al., 2010), personality characteristics (Buhrmester et al., 2011), cognitive biases (Paolacci et al., 2010), and mental health measures (Shapiro et al., 2013). Participants’ ages ranged from 18 to 66 years (*M* = 35.50, *SD* = 10.67).

#### Procedure and Materials

Participants were compensated $1 for completing a 10 min online questionnaire consisting of a loneliness questionnaire, a nostalgia inventory, and two measures of approach-related social goals/intentions.

##### Loneliness

The 10-item UCLA loneliness questionnaire (Russell, 1996) described in Study 1 was used to assess loneliness (α = 0.93; *M* = 2.17, *SD* = 0.65).

##### Nostalgia

The state version of the Nostalgia Inventory (Batcho, 1995) described in Study 1 was used as a measure nostalgic feelings (α = 0.92; *M* = 4.17, *SD* = 1.29).

##### Approach-related social goals/intentions

The questionnaire included two measures meant to assess approach-related social goals/intentions. First, Elliot et al. (2006) 4-item friendship-approach goal scale was used to assess approach-related social goals. The items assess the extent to which people are committed to goals related to interpersonal gains and growth (e.g., “I feel that I want to move toward growth and development in my friendships”; 1 = *strongly disagree*, 6 = *strongly agree*; α = 0.92; *M* = 4.31, *SD* = 1.13).

Second, Abeyta et al. (2015a) friendship conflict task was used to assess intentions for resolving conflict. The friendship conflict task instructs participants to imagine a conflict with their best friend and then responded to three items on their intentions to be about resolving the conflict (e.g., “I would dedicate myself to solving this conflict”; 1 = *strongly disagree*, 6 = *strongly agree*; α = 0.91; *M* = 4.65, *SD* = 1.16).

### Results

First, we conducted correlations between the measured variables. Loneliness was significantly and negatively correlated with approach-oriented goals and intentions for resolving social conflict. State nostalgia was significantly and positively associated with approach-oriented goals and intentions for resolving friendship conflict. Loneliness was negatively correlated with state nostalgia, which is again inconsistent with the mediation model of loneliness predicting increased nostalgia. These correlations can be found in Table 1.

#### Approach-Oriented Friendship Goals

Next, we conducted hierarchical linear regression analyses regressing approach-oriented friendship goals on state nostalgia (centered at the mean) and loneliness (centered at the mean) in the first step. In the second step, we added the state nostalgia × loneliness interaction. This overall equation was significant, *R*^2^ = 0.42, *F*(3, 196) = 35.75, *p* < 0.001.

A regression analysis revealed a significant main effect of loneliness on approach-oriented social goals, such that greater loneliness was associated with lower levels of social approach, *b* = −0.90, *SE* = 0.10, *t* = −9.17, *p* < 0.001, *sr*^2^ = 0.27, 95% CI [−1.10, −0.71]. There was also a significant main effect of state nostalgia, such that greater feelings of nostalgia were associated with greater social approach, *b* = 0.22, *SE* = 0.05, *t* = 4.42, *p* < 0.001, *sr*^2^ = 0.06, 95% CI [0.12, 0.32]. These main effects were qualified by a significant loneliness × state nostalgia interaction, *b* = 0.25, *SE* = 0.06, *t* = 4.07, *p* < 0.001, *sr*^2^ = 0.05, 95% CI [0.13, 0.37].

**FIGURE 2:**
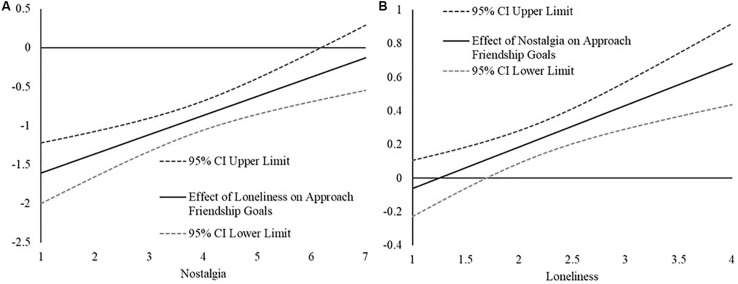
**(A)** The plot represent; the estimated relation between loneliness and approach-oriented friendship goals (*y*-axis) across the range of nostalgic feeling; (*x*-axis) in Study 2. The relation; are considered statistically significant if the confidence intervals for the effect (Cl) do not contain 0. **(B)** The represents the estimated relation between nostalgic feelings and approach- oriented friendship goals (*y*-axis) across the range of loneliness scores (*x*-axis) in Study 2. The relations are considered statistically significant if the confidence intervals for the effect (Cl) do not contain 0.

As in Study 1, probed the interaction with the Johnson and Neyman (1936) technique. First, we examined how the relation between loneliness and approach-oriented friendship goals changed as a function of nostalgia. As can be seen in Figure 2A, the strength of the loneliness and approach-oriented friendship goals relation weakens (i.e., becomes less strongly negative) as a function of greater state nostalgia and becomes non-significant at very high levels (greater than 6.17, between 1 and 2 SD above the mean) of state nostalgia.

For the sake of being exhaustive, we also examined the relation between nostalgia and approach-oriented friendship goals as a function of loneliness. As is seen in Figure 2B, the Johnson and Neyman (1936) technique revealed that the relation between nostalgia and approach-oriented friendship goals became more strongly positive as a function of greater loneliness, becoming statistically significant at moderate levels of loneliness (1.75, between −1 SD and the mean).

#### Intentions for Overcoming Social Conflict

We conducted hierarchical linear regression analyses regressing intentions for overcoming social conflict on state nostalgia (centered at the mean) and loneliness (centered at the mean) in the first step. In the second step, we added the state nostalgia × loneliness interaction. This overall equation was significant, *R*^2^ = 0.26, *F*(3, 196) = 24.31, *p* < 0.001.

A regression analysis revealed a significant main effect of loneliness on intentions for resolving a social conflict, such that greater loneliness was associated with lower levels of intentions, *b* = −0.71, *SE* = 0.11, *t* = −6.29, *p* < 0.001, *sr*^2^ = 0.16, 95% CI [−0.93, −0.49]. There was also a significant main effect of state nostalgia, such that greater feelings of nostalgia were associated with more intentions for resolving a social conflict, *b* = 0.19, *SE* = 0.06, *t* = 3.38, *p* = 0.001, *sr*^2^ = 0.04, 95% CI [0.08, 0.31]. These main effects were qualified by a significant loneliness × state nostalgia interaction, *b* = 0.24, *SE* = 0.07, *t* = 3.37, *p* = 0.001, *sr*^2^ = 0.04, 95% CI [0.10, 0.37].

**FIGURE 3:**
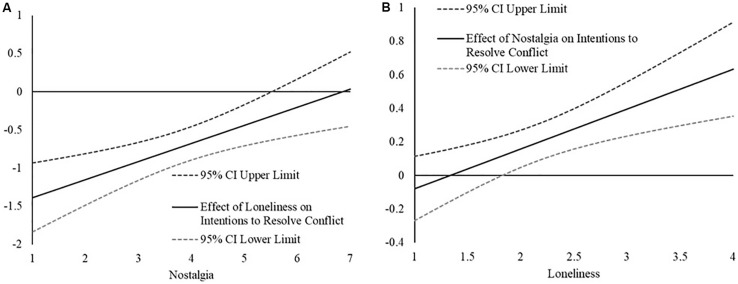
**(A)** The plot represents the estimated relation between loneliness and intentions for overcoming social conflict (*y*-axis) across the range of nostalgic feelings (*x*-axis) in Study 2. The relations are considered statistically significant if the confidence intervals for the effect (CI) do not contain 0. **(B)** The plot represents the estimated relation between nostalgic feelings and intentions for overcoming social conflict (*y*-axis) across the range of loneliness scores (*x*-axis) in Study 2. The relations are considered statistically significant if the confidence intervals for the effect (Cl) do not contain 0.

First, we examined how the relation between loneliness and intentions for resolving social conflict changed as a function of nostalgia. As can be seen in Figure 3A, the Johnson and Neyman (1936) technique revealed that the strength of the loneliness and intentions relation weakens (i.e., become less strongly negative) as a function of greater state nostalgia and becoming non-significant at very high levels (greater than 5.50, slightly less than 1 *SD* above the mean) of state nostalgia.

For the sake of being thorough, we also examined how the relation between nostalgia and intentions for resolving social conflict as a function of loneliness. As can be seen in Figure 3B, the Johnson and Neyman (1936) technique revealed that the relation between nostalgia and intentions for resolving social conflict became more strongly positive as a function of greater loneliness, becoming statistically significant at low to moderate levels of loneliness (1.90, between −1 SD and the mean).

Taken together, the results of Study 2 provide evidence that the tendency for lonely people to be less oriented toward approach-oriented social goals/intentions is weaker when people report feeling nostalgic. Specifically, the association between loneliness and lower social approach goal commitment was strongest at low levels of state nostalgia but was found to be weaker at higher levels of nostalgia. Similarly, the association between loneliness and a reduced desire to be in resolving a friendship conflict was found to be weaker as a function of increases in nostalgia. This weakening appeared to be explained by nostalgia’s positive association with approach-oriented goals and intentions. Approach-oriented social goals and intentions are relatively high at low levels of loneliness and do not vary as a function of nostalgic feelings, whereas at higher levels of loneliness, individuals who report stronger nostalgic feelings have less of a deficit in approach goals and intentions.

## Study 3

The purpose of Study 3 was to replicate and extend the Study 2 findings. As in Study 2, we assessed trait loneliness, state nostalgia, and approach-related social goals. We expected nostalgia to moderate the relation between loneliness and commitment to approach-related social goals in the same manner as in Study 2. To extend the Study 2 findings, we included a more behavioral measure of approach-related social goal commitment: willingness to participate in upcoming research studies that do or do not involve social interaction. Once again, we hypothesized that that the negative relation between loneliness and willingness to participate in social research would be weaker as a function of nostalgia, because nostalgia would be positively associated with willingness to participate in social research, particularly at higher levels of loneliness. Past research indicates that nostalgia broadly promotes approach-oriented motivations (e.g., Stephan et al., 2014). The inclusion of the non-social research study option allowed us to explore whether the tendency for nostalgia to moderate the relation between trait-loneliness and approach-oriented social goals extends to approach-oriented goals that are not social. Consistent with Abeyta et al. (2015a) finding that nostalgia increased intentions to participate in social but not non-social research studies, we did not expect nostalgia to moderate the relationship between loneliness and intentions to participate in a non-social research study.

### Materials and Methods

#### Participants

Participants consisted of 181 undergraduate students from a Midwestern university (79 female participants). Participants’ ages ranged from 18 to 35 years (*M* = 19.08, *SD* = 1.91).

#### Procedure

The study consisted of a computerized questionnaire. Participants completed all materials on computers in private cubicles and were fully debriefed after the study session.

#### Materials

##### Loneliness

As in Studies 1 and 2, participants completed the 10-item UCLA loneliness questionnaire (Russell, 1996; α = 0.90; *M* = 2.11, *SD* = 0.56).

##### Nostalgia

Participants completed the state nostalgia measure used in Studies 1 and 2 (Batcho, 1995; α = 0.88; *M* = 4.86, *SD* = 0.95).

##### Approach-related social goals

Elliot et al. (2006) 4-item friendship-approach goal scale used in Study 2 was used to assess approach-related social goals (α = 0.86; *M* = 5.38, *SD* = 1.05).

##### Future study participation task

Participants were told that one of the purposes of the study is to gauge their interest in, and to promote, future research studies. Next, participants read a description of two studies they could participate in Abeyta et al. (2015a). One of the research studies is social in nature, whereas the other study is non-social. The social study is titled “Personality and Social Interaction” and is described as a study where participants get to know a stranger by having discussions about predetermined topics. The non-social study is titled “Cognitive Problem Solving” and is described as a study testing individuals’ ability to solve complex puzzles. Participants indicated (1) how interested they would be to participate in each study (1 = *not interested*, 7 = *very interested*), (2) whether or not they would like to learn more information about each study (1 = *definitely no*, 7 = *definitely yes*), and (3) whether or not they would like to participate in each study (1 = *definitely no*, 7 = *definitely yes*). Scores were computed for willingness to participate in the social research study (α = 0.95; *M* = 4.91, *SD* = 1.59) and willingness to participate in the non-social research study (α = 0.96; *M* = 4.81, *SD* = 1.79), respectively.

### Results and Discussion

First, we conducted correlations between the measured variables. Loneliness was significantly and negatively correlated with approach-oriented goals but was not significantly correlated with intentions for participating in social and non-social research. State nostalgia was significantly and positively associated with approach-oriented goal and participation in non-social research studies, but was not significantly correlated with participation in social research studies. Loneliness was not significantly correlated with state nostalgia, again, inconsistent with a mediation model approach. These correlations can be found in Table 1.

#### Approach-Oriented Social Goals

Next, we conducted hierarchical linear regression analyses regressing approach-oriented friendship goals on state nostalgia (centered at the mean) and loneliness (centered at the mean) in the first step. In the second step, we added the state nostalgia × loneliness interaction. This overall equation was significant, *R*^2^ = 0.17, *F*(3, 177) = 13.35, *p* < 0.001.

A regression analysis revealed a significant main effect of loneliness on approach-oriented social goals, such that greater loneliness was associated with lower levels of social approach, *b* = −0.50, *SE* = 0.13, *t* = −3.92, *p* < 0.001, *sr*^2^ = 0.07, 95% CI [−0.76, −0.25]. There was also a significant main effect of state nostalgia, such that greater feelings of nostalgia were associated with greater social approach, *b* = 0.30, *SE* = 0.08, *t* = 3.96, *p* < 0.001, *sr*^2^ = 0.08, 95% CI [0.15, 0.45]. These main effects were qualified by a significant loneliness × state nostalgia interaction, *b* = 0.39, *SE* = 0.14, *t* = 2.86, *p* = 0.005, *sr*^2^ = 0.04, 95% CI [0.12, 0.66].

First, we examined how the relation between loneliness and approach-oriented friendship goals changed as a function of nostalgia. Replicating Study 2, the Johnson and Neyman (1936) revealed that the strength of the loneliness and approach-oriented friendship goals relation weakens (i.e., becomes less strongly negative) as a function of greater state nostalgia and becomes non-significant at higher levels (greater than 5.50, between the mean and 1 *SD*) of state nostalgia.

For the sake of being thorough, we also examined the relation between nostalgia and approach-oriented friendship goals as a function of loneliness. The Johnson and Neyman (1936) technique revealed that the relation between state nostalgia and approach-oriented friendship goals became more strongly positive as a function of greater loneliness, becoming statistically significant at low to moderate (1.81, between −1 SD and the mean) levels of loneliness.

#### Participation in Social and Non-social Research Studies

We conducted hierarchical linear regression analyses regressing intentions to participate in social research studies on state nostalgia (centered at the mean) and loneliness (centered at the mean) in the first step. In the second step, we added the state nostalgia × loneliness interaction. This overall equation was significant, *R*^2^ = 0.03, *F*(3, 177) = 2.73, *p* = 0.046.

The main effects of loneliness, *b* = −0.16, *SE* = 0.21, *t* = −0.77, *p* = 0.44, *sr*^2^ = 0.003, 95% CI [−0.58, 0.25], and nostalgia, *b* = 0.13, *SE* = 0.12, *t* = 1.03, *p* = 0.31, *sr*^2^ = 0.006, 95% CI [−0.12, 0.37], on willingness to participate in the social research study did not reach statistical significance. However, the loneliness × state nostalgia interaction was statistically significant, *b* = 0.57, *SE* = 0.22, *t* = 2.55, *p* = 0.01, *sr*^2^ = 0.03, 95% CI [0.13, 1.01].

**FIGURE 4:**
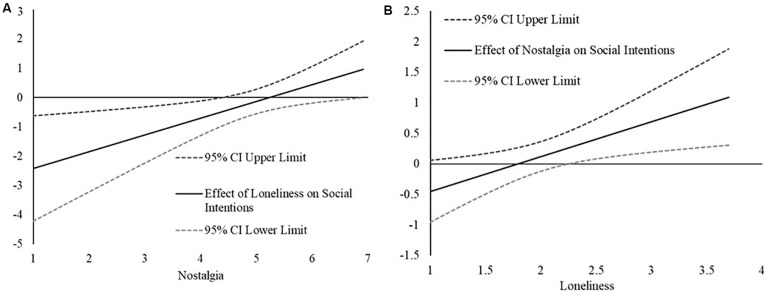
**(A)** The plot represents the estimated relation between loneliness and intentions for participating in social research (*y*-axis) across the range of nostalgic feelings (*x*-axis) in Study 3. The relations are considered statistically significant if the confidence intervals for the effect (CI) do not contain 0. **(B)** The plot represents the estimated relation between nostalgic feelings and social intentions (*y*-axis) across the range of loneliness scores (*x*-axis) in Study 3. The relations are considered statistically significant if the confidence intervals for the effect (CI) do not contain 0.

First, we examined how the relation between loneliness and approach-oriented friendship goals changed as a function of nostalgia. As can be seen in Figure 4A, the Johnson and Neyman (1936) technique revealed that the association between loneliness and intentions to participate in social research relation becomes less strongly negative as a function of greater state nostalgia, becoming non-significant at moderate levels (greater than 4.40, slightly below the mean) of state nostalgia.

We also examined how the relation between nostalgia and intentions to participate in social research as a function of loneliness. As can be seen in Figure 4B, the Johnson and Neyman (1936) technique revealed that the relation between nostalgia and intentions to participate in social research became more strongly positive as a function of greater loneliness, becoming statistically significant at moderate (2.35, slightly above the mean) levels of loneliness.

Next, we conducted hierarchical linear regression analyses regressing willingness to participate in non-social research on state nostalgia (centered at the mean), loneliness (centered at the mean), and the state nostalgia × loneliness interaction. This overall equation was significant, *R*^2^ = 0.03, *F*(3, 177) = 2.61, *p* = 0.05.

A regression analyses revealed that the main effect of loneliness on willingness to participate in non-social research was not statistically significant, *b* = 0.37, *SE* = 0.23, *t* = 1.57, *p* = 0.12, *sr*^2^ = 0.01, 95% CI [−0.09, 0.83]. There was a significant main effect of state nostalgia, such that greater nostalgia was associated with greater willingness to participate in the non-social research study, *b* = 0.28, *SE* = 0.14, *t* = 2.03, *p* = 0.04, *sr*^2^ = 0.02, 95% CI [0.008, 0.55]. The loneliness × state nostalgia interaction, *b* = −0.27, *SE* = 0.25, *t* = −1.09, *p* = 0.28, *sr*^2^ = 0.006, 95% CI [−0.77, 0.22], did not reach statistical significance.

The results of Study 3 replicate and extend the Study 2 findings using a more behavioral measure of approach-related social intentions; participants’ willingness to participate in an upcoming research study that involves social interaction. Loneliness was associated with reduced desire to sign-up for a research study involving social interaction. However, this tendency was found to be weaker when high levels of nostalgia were reported, because nostalgia, particularly at higher levels of loneliness, was positively associated with desire to sign up for social research studies. Finally, the observed main effect of nostalgia on willingness to participate in a non-social study is consistent with the broader literature indicating that nostalgia increases approach-oriented goals and behavior (Stephan et al., 2014).

## Study 4

The purpose of Study 4 was to provide vital causal evidence for the patterns of data observed across the three previous non-experimental studies. We accomplished this by inducing high v low levels of loneliness, then having people reflect on a nostalgic or non-nostalgic memory, and finally assessing approach-oriented social intentions. Once again, we expected nostalgia to moderate the effect of loneliness on commitment to approach-oriented social intentions. Consistent with Studies 2 and 3, we expected loneliness to decrease approach-oriented social intentions in a control condition (i.e., when people reflect on a non-nostalgic memory) but that nostalgia would reduce this effect. Moreover, we once again hypothesized that this effect would be driven by nostalgia’s association with increased approach-oriented social intentions, particularly at high levels of loneliness.

### Materials and Methods

#### Participants

A total of 400 participants residing in the United States (175 female participants) were recruited from AMT (*M*_*age*_ = 36.09, *SD*_*age*_ = 10.61). Recent evidence indicates that AMT participants tend to be familiar with the use of deception in psychological research and that this non-naiveté can undermine validity of experimental paradigms (for a review see, Hauser et al., 2019). We thought that it was important to address this concern for the current research, because we used a false feedback paradigm to manipulate loneliness (see description in Materials section). Moreover, recently there have been a number of concerns about low-quality AMT responses from automated programs and/or non-English-speaking AMT workers outside of the United States using duplicate geolocations and server farms to complete research (Bai, 2018; Moss and Litman, 2018, Sept. 18). Due to these concerns, we developed a plan for identifying and excluding non-naïve and suspicious low-quality open-ended responses.^1^ In total, 129 cases were excluded from the analyses: 106 cases that believed the loneliness feedback was fake and 23 for having low-quality open-ended responses. This left a final sample of 271 (122 women) AMT workers (*M*_*age*_ = 35.61, *SD*_*age*_ = 10.18).

#### Procedure

Participants were compensated $1 for completing an online questionnaire that consisted of a loneliness manipulation task, a nostalgia or control manipulation, and a measure of approach-oriented social intentions.

#### Materials

##### Loneliness manipulation task

We adapted a false-feedback paradigm used by Wildschut et al. (2006) to manipulation loneliness. In particular, participants were randomly assigned to a low loneliness or a high loneliness condition. In both conditions, participants completed 10 items from the UCLA loneliness questionnaire (Russell, 1996) and were told that they would receive feedback on their score afterward. In the low loneliness condition, items were worded elicit disagreement (e.g., “I always feel left out,” 1 = *strongly disagree*, 4 = *strongly agree*), and after completing the loneliness questionnaire participants were given their actual score and the following feedback: “Your loneliness scores is [participants summed score]. This score is in the 12th percentile of people in the United States. This means your level of loneliness is very low.” In the high loneliness condition, items were worded to elicit agreement (e.g., “I sometimes feel left out,” 1 = *strongly disagree*, 4 = *strongly agree*), and participants were given their actual score and the following feedback: “Your loneliness scores is [participants summed score]. This score is in the 67th percentile of people in the United States. This means your level of loneliness is well above average.”

##### Memory reflection task

We used a version of the event reflection task to manipulate nostalgia (Wildschut et al., 2006). Specifically, participants were randomly assigned to a nostalgia or control condition. In the nostalgia condition, participants were presented with the nostalgia definition that accompanied the nostalgia inventory in Studies 1–3 and were instructed to bring to mind and write about a nostalgic memory. In the control condition, participants were instructed to bring to mind and write about any memory and were not presented with a definition of or any information about nostalgia.

##### Future study participation task

Participants completed the study participation task (Abeyta et al., 2015a) described in Study 3. Participants indicated (1) how interested they would be to participate in the study (1 = *not interested*, 7 = *very interested*), (2) whether or not they would like to learn more information about the study (1 = *definitely no*, 7 = *definitely yes*), and (3) whether or not they would like to participate in the study (1 = *definitely no*, 7 = *definitely yes*) in a social research study (α = 0.97; *M* = 5.16, *SD* = 2.00) and non-social research study (α = 0.96; *M* = 6.00, *SD* = 1.48), respectively.

### Results

**FIGURE 5:**
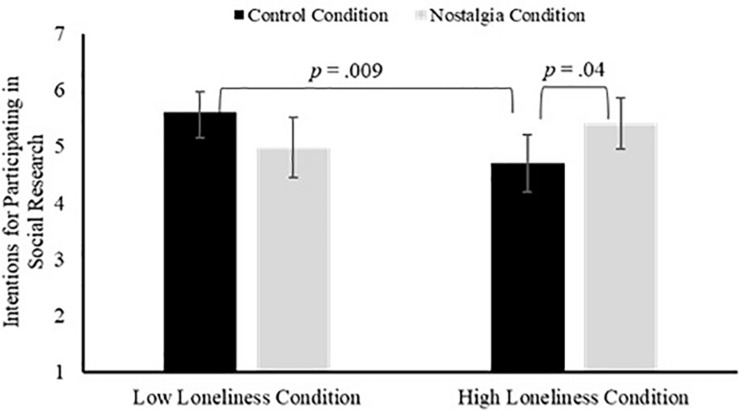
The plot represents the effects of loneliness and nostalgia on intentions to participate in social research studies in Study 4. Significant differences are labeled with relevant *p*-value.

We analyzed the data with 2 × 2 between-subjects ANOVA to determine the unique effects of the loneliness and nostalgia manipulations and their combined effect on willingness to participate in social research. The main effects of loneliness, *F*(1, 267) = 0.80, *p* = 0.37, η*_*p*_*^2^ = 0.003, and nostalgia, *F*(1, 267) = 0.07, *p* = 0.79, η*_*p*_*^2^ < 0.001, did not reach statistical significance. However, the hypothesized loneliness × nostalgia interaction did reach statistical significance, *F*(1, 267) = 7.29, *p* = 0.007, η*_*p*_*^2^ = 0.03. See Figure 5 for a graph of the interaction.

To probe the interaction, we first conducted two *t*-tests to examine the effect of loneliness (high v low) within the nostalgia condition and the control condition, respectively. In the control condition, participants in the high loneliness condition reported significantly lower interest in participating in social research than did participants in the low loneliness condition, *t*(131) = −2.64, *p* = 0.009, *d* = 0.46. In the nostalgia condition, the difference between the high loneliness condition and the low loneliness condition on participation in social research did not reach statistical significance, *t*(131) = 1.24, *p* = 0.22, *d* = 0.21. Next, we conducted two *t*-tests to examine the effect of the nostalgia compared to the control condition within the low loneliness condition and the high loneliness condition, respectively. In the low loneliness condition, the difference between the nostalgia and control condition did not reach statistical significance, *t*(131) = −1.72, *p* = 0.09, *d* = 0.30. However, in the high loneliness condition, participants in the nostalgia condition reported greater interest in participating in social research than did participants in the control condition (*M* = 4.70, *SD* = 2.07), *t*(131) = 2.10, *p* = 0.04, *d* = 0.36.

Next, we conducted a 2 × 2 between-subjects ANOVA to determine the unique effects of the loneliness and nostalgia manipulations and their combined effect on willingness to participate in non-social research. The main effects of loneliness, *F*(1, 267) = 0.19, *p* = 0.66, η*_*p*_*^2^ = 0.001, and nostalgia, *F*(1, 267) = 2.60, *p* = 0.11, η*_*p*_*^2^ = 0.01, and the loneliness × nostalgia interaction, *F*(1, 267) = 0.13, *p* = 0.72, η*_*p*_*^2^ < 0.001, did not reach statistical significance.

Study 4 provided experimental evidence replicating the non-experimental evidence in the previous studies. Specifically, in a control condition in which participants brought to mind any memory, the loneliness manipulation decreased intentions to participate in a future research that involved social interaction. Nostalgia appeared to mitigate this effect. In the nostalgia condition in which participants brought to mind a nostalgic memory, the effect of the loneliness manipulation on intentions to participate in a future research that involved social interaction was small and not statistically significant. This effect was driven by nostalgia’s capacity to promote approach-oriented social goals/intentions, particularly at high levels of loneliness. Nostalgia appeared to increase intentions to participate in a future research study that involved social interaction in the high loneliness condition, but not in the low loneliness condition. Finally, Study 4 provided experimental evidence that the effect of loneliness and nostalgia may be specific to the social domain, because there were no significant effects on intentions to participate in non-social research.

## General Discussion

Though past research has demonstrated that people turn to nostalgia when feeling lonely (Wildschut et al., 2006) and that nostalgia in turn regulates loneliness by promoting feelings of social support (Zhou et al., 2008), the current research was the first to provide evidence supporting nostalgia’s potential to regulate lack of social-confidence and negative motivational tendencies associated with loneliness. Study 1 provided evidence that the inverse relation appeared to be weaker as a function of stronger nostalgic feelings, because nostalgic feelings were associated with greater social confidence, particularly at high levels of loneliness. Thus, recruiting nostalgia may mitigate the tendency for lonely people to lack confidence in their abilities to establish meaningful bonds. Studies 2, 3, and 4 built upon this finding by focusing more specifically on interpersonal goals/intentions. Studies 2 and 3 provided non-experimental evidence that nostalgia moderated the inverse relations between loneliness and approach-related friendship goals (Studies 2 and 3), loneliness and intentions for resolving friendship conflict (Study 2), and loneliness and intentions to engage in a social interaction (Study 3), respectively. Once again, these weaker inverse associations at high levels of nostalgia appeared to be due to nostalgia’s positive association with approach-oriented social goals/intentions. Study 4 provided experimental evidence that nostalgia mitigates the negative relation between loneliness and a reduced social approach orientation. Specifically, manipulated loneliness decreased intentions to engage in a social interaction in a control condition in which people brought to mind an unspecified memory, but loneliness did not affect intentions to engage in a social interaction when people brought to mind a nostalgic memory. Moreover, the mitigated effect appears to be due to nostalgia’s capacity to energize approach-oriented social goals/intentions, particularly at high levels of loneliness. Specifically, nostalgia increased intentions to engage in a social interaction when people were made to feel lonely.

The current findings build on recent research supporting the social cognitive and motivational benefits of nostalgia. In terms of the social cognitive benefits, past research has found that experimentally manipulated nostalgia increases feelings of social confidence/efficacy (Wildschut et al., 2006; Abeyta et al., 2015a) and generally promotes a positive future outlook (e.g., Cheung et al., 2013; Abeyta and Routledge, 2016). The findings of Study 1 complement this past research, demonstrating that feelings of nostalgia are associated with stronger social confidence. In terms of motivational benefits, past research has linked the propensity for engaging in nostalgic reverie to increased approach motivation (Stephan et al., 2014) and has found that experimentally evoked nostalgia increases approach-related social goals and intentions (Stephan et al., 2014; Abeyta et al., 2015a). The findings of Studies 2 and 3 complement these, demonstrating that feelings of nostalgia are associated with greater approach-related goals and intentions. Moreover, Study 4 provided evidence that engaging in nostalgic reveries promotes approach-related orientation, particularly when people are made to feel lonely. Taken together, the current package of studies extends this past research on the social cognitive and motivational benefits of nostalgia by providing evidence that nostalgia regulates negative social cognitions and motivational tendencies typical of chronically lonely individuals.

In the present research, loneliness was not strongly related to state nostalgia; trait loneliness was either uncorrelated with state nostalgia (Study 3) or was negatively, but weakly (Studies 1 and 2), correlated with state nostalgia. In contrast, past research (Zhou et al., 2008) has evidenced positive associations between loneliness and trait nostalgia (i.e., nostalgia proneness). This discrepancy may be resolved by considering the distinction between trait and state nostalgia. Chronically lonely people are more likely to experience situations that trigger feelings of loneliness or social exclusion (Hanley-Dunn et al., 1985), and research has found that experiences that trigger lonely feelings bring on line nostalgic feelings (Wildschut et al., 2006; Zhou et al., 2008). In general, however, chronically lonely individuals tend to ruminate on negative social experiences and their social inadequacies instead of longing for positive social memories, especially when reminders of social belonging deficits are lacking (Anderson et al., 1983; Vanhalst et al., 2015). Nostalgic memories typically involve reminiscing on cherished social experiences (e.g., Abeyta et al., 2015a), and thus chronically lonely people may feel less nostalgic than non-chronically lonely in the absence of loneliness triggers. Alternatively, the lack of association between loneliness and nostalgia may also explained by an individual difference, like psychological resilience, that we did not consider. Indeed, Zhou et al. (2008) found that loneliness was associated with greater trait nostalgia among individuals high but not low in psychological resilience. Ultimately, our goal was to focus on whether the experience of nostalgia moderates the relation between loneliness and negative intrapsychic tendencies generally, so we did not account for individual differences. This was a limitation of the current research, and so future research should explore potential moderating individual difference variables to further understand when lonely people turn to nostalgia.

The current package of studies has broad implications for the health ramifications of chronic loneliness. As we discussed in the opening of the manuscript, chronic loneliness is a very serious risk factor for mental illness and physical disease (Cacioppo and Cacioppo, 2014). Therefore, it is important to identify interventions to alleviate it. Combined with past research that nostalgia regulates loneliness by affirming social support (Zhou et al., 2008), the current research contributes to understanding nostalgia’s intervention potential. Meta-analyses of loneliness interventions indicate that interventions that solely target social support are not strong enough to effectively overcome chronic loneliness. In contrast, interventions that include techniques like cognitive behavioral therapy (CBT) that target maladaptive social cognitions associated with loneliness tend to be more effective than interventions that seek to reduce loneliness by solely focusing on bolstering social support, teaching social skills, or increasing the availability of social interactions. This is likely because loneliness is a subjective feeling of aloneness that is based on the feeling that an individual’s interpersonal relationships are unfulfilling, irrespective of whether an individual is objectively socially isolated (Masi et al., 2011; Cacioppo et al., 2015). Thus, any successful intervention should focus on the thoughts and feelings individuals have about their interpersonal relationships. Past research suggests that nostalgia can be effective as part of a loneliness intervention by promoting perceptions of social support (Zhou et al., 2008). The current findings take our understanding of nostalgia’s intervention potential further, by suggesting that nostalgia can help to regulate the negative social cognitions and motivational tendencies typical of lonely individuals. However, an important limitation of the current research is that our sample did not score particularly high on loneliness and consisted of internet samples. Future research should look to replicate out findings with community and/or clinical samples of people being treated for chronic loneliness. Another limitation of the current research was that we only considered two maladaptive intrapsychic tendencies typical of chronic loneliness, namely, deficits in social confidence and reduced approach-oriented social goals. Research has found that there are other maladaptive intrapsychic tendencies associated with chronic loneliness that make reversing loneliness difficult. For example, research indicates that chronically lonely individuals are more apt to focus on and be impacted by negative social interactions, compared to positive social interactions. This tendency to focus on negative social experiences and downplay or ignore positive social experiences makes overcoming loneliness difficult (Vanhalst et al., 2015). Future research should also consider whether nostalgia mitigates these negative emotional biases. The emotional impact of nostalgia has been found to be overwhelmingly more positive than negative (e.g., Wildschut et al., 2006; Abeyta et al., 2015b) and nostalgia has been found to instigate optimism (Cheung et al., 2013) so it may be particularly suited to also help overcome these negative emotional biases. Finally, future research should also consider utilizing experiences that promote nostalgic feelings as part of loneliness interventions. Used in conjunction with traditional interventions like CBT, nostalgia may foster a sense of social support and help equip people with the intrapsychic tools to connect with others. Based on the current research, stopping the cycle of loneliness may involve looking to the past for confidence and encouragement.

## Data Availability Statement

The datasets generated for this study are available on request to the corresponding author.

## Ethics Statement

The studies involving human participants were reviewed and approved by the Rutgers University Arts and Science IRB North Dakota State University Institutional Review Board. The patients/participants provided their written informed consent to participate in this study.

## Author Contributions

AA wrote the introduction, discussion, results sections, played 60% role in conception, study design, and collection of data. CR provided feedback on drafts of the manuscript and played 40% role in conception, study design, and collection of data. SK wrote the method sections for Studies 1–3. All authors contributed to the article and approved the submitted version.

## Conflict of Interest

The authors declare that the research was conducted in the absence of any commercial or financial relationships that could be construed as a potential conflict of interest.

## Footnotes

1 To guard against non-naïve participants, we included the following open-ended probing question: “What were your thoughts on the loneliness feedback you received?” Prior to collecting the data, we made a plan to review participant responses and exclude any participants who specifically mentioned that they did not believe the feedback they received. Examples of excluded cases include: “I thought it was completely made up,” “I knew it was fake because I’m extremely lonely on the scale and it said I wasn’t,” “I felt it was inaccurate”). We also planned on reviewing participant responses to the open-ended nostalgia or control writing task to screen for low-quality responses. Specifically, we planned on excluding “suspicious cases.” We defined a “suspicious case” broadly as any response to the nostalgia or control task that did not follow the writing prompt and/or did not make sense. In our review, we found that a number of responses were identical. For example, 6 suspicious cases (5 in the nostalgia condition and 1 in the control condition) contained the following response: “The adjective nostalgic is often used to describe someone who is homesick…” Moreover, there were two instances where two cases had identical memory task responses and identical responses to the loneliness feedback prompt. There were also two cases where written responses appear to have been copied from articles from the internet. The other 11 cases had phrases such as “good,” “very feel,” and “good and nice” for the memory writing task and loneliness probing question. All “suspicious cases” and cases that expressed not believing the loneliness feedback were excluded before conducting analyses.
