# Supplementary material for: Combating Loneliness With Nostalgia: Nostalgic Feelings Attenuate Negative Thoughts and Motivations Associated With Loneliness
Source: Front Psychol. 2020 Jun 23;11:1219. doi: 10.3389/fpsyg.2020.01219 (PMC7324708; doi:10.3389/fpsyg.2020.01219)
Supplement: Supplementary file 1 [file Data_Sheet_1.docx]

**Supplementary Materials**

**Trait Loneliness (Studies 1-3)**

*Instructions:* The following statements describe how people sometimes feel. For each statement, please indicate how often you feel the way described.

NEVER RARELY SOMETIMES ALWAYS

1 2 3 4

1. How often do you feel that you lack companionship?

2. How often do you feel that you have a lot in common with the people around you?

3. How often do you feel close to people?

4. How often do you feel left out?

5. How often do you feel that no one really knows you well?

6. How often do you feel isolated from others?

7. How often do you feel that there are people who really understand you?

8. How often do you feel that people are around you but not with you?

9. How often do you feel that there are people you can talk to?

10. How often do you feel that there are people you can turn to?

Citation:

Russell, D. W. (1996). UCLA Loneliness Scale (version 3): Reliability, validity, and factor structure. *Journal of Personality Assessment, 66,* 20-40.

**State Nostalgia (Studies 1-3)**

According to the Oxford Dictionary, ‘nostalgia’ is defined as a ‘sentimental longing for the past.’
This questionnaire is designed to measure what you are feeling **AT THIS MOMENT**. Please indicate how nostalgic you feel about each of the 20 persons, situations, or events below.

| I am not very nostalgic about |  |  |  |  |  | I am very nostalgic about |
| --- | --- | --- | --- | --- | --- | --- |
| 1 | 2 | 3 | 4 | 5 | 6 | 7 |

| 1. My family | 11. Feelings I had |
| --- | --- |
| 2. Vacations I went on | 12. My school |
| 3. Places | 13. Having someone to depend on |
| 4. Music | 14. Not having to worry |
| 5. Somone I loved | 15. The way society was |
| 6. My friends | 16. My pets |
| 7. Things I did | 17. Not knowing sad or evil things |
| 8. My childhood toys | 18. TV shows, movies |
| 9. The way people were | 19. My family house |
| 10. My heroes/heroines | 20. My church/religion |

Citation:

Batcho, K. I. (1995). Nostalgia: A psychological perspective. *Perceptual and Motor Skills, 80,* 131-143.

**Social-Efficacy (Study 1)**

Based on how you are feeling right now, please rate how certain you are that you can do the things described. Rate the degree of confidence by selecting a number from 1 to 10.

| 1 | 2 | 3 | 4 | 5 | 6 | 7 | 8 | 9 | 10 |
| --- | --- | --- | --- | --- | --- | --- | --- | --- | --- |
| cannot do at all |  |  |  |  |  |  |  |  | highly certain can do |

1. Establish successful social relationships.

2. Maintain social relationships.

3. Resolve conflicts in social relationships.

4. Communicate effectively in social relationships.

5. Open up to others in social relationships.

6. Approach people I don’t know and strike up a conversation.

Citation:

Bandura, A. (2006). Guide for constructing self-efficacy scales. In F. Pajares, & T. Urdan (Eds.), *Self-efficacy beliefs of adolescents* (pp. 307-337). Greenwich, CT: Information Age Publishing.

**Approach-Oriented Social Goals (Studies 2 & 3)**

Please indicate the extent to which the goals below describe your goals/motivations.

| 1 | 2 | 3 | 4 | 5 | 6 | 7 |
| --- | --- | --- | --- | --- | --- | --- |
| not very true of my current feelings |  |  |  |  |  | very true of my current feelings |

I am….

1. Trying to deepen my relationships with my friends.

2. Trying to move toward growth and development in my friendships.

3. Trying to enhance the bonding and intimacy in my close relationships.

4. Trying to share many fun and meaningful experiences with my friends.

Citation :

Elliot, A. J., Gable, S. L., & Mapes, R. R. (2006). Approach and avoidance motivation in the social domain. Personality and Social Psychology Bulletin, 32, 378-391.

**Proactive Intentions for Resolving Friendship Conflict (Study 2)**

*Instructions:* For this next task bring to mind a close friend of yours. This might be someone you would describe as your best friend, but if not should be someone you have a close relationship with and have known for a while.

**Please write your friends first name in the box below.**

|  |
| --- |

Now imagine that you and your close friend got into a disagreement. You and your friend have tried to resolve this conflict, but things just are not the same. You have noticed that since the disagreement, you and your friend hangout less often. When you do see your friend he/she seems a bit cold and distant. Sure your friend is nice enough and you get along, but it is clear this disagreement has driven a wedge between you.

Consider how a disagreement between you and your close friend would make you feel and respond to each question. Please answer the following questions based on how you are feeling **right now** and not your general attitudes.

1. I would dedicate myself to solving this conflict.

| 1 | 2 | 3 | 4 | 5 | 6 |
| --- | --- | --- | --- | --- | --- |
| strongly disagree | moderately disagree | slightly disagree | slightly agree | moderately agree | strongly agree |

2. I would be proactive in solving this conflict.

| 1 | 2 | 3 | 4 | 5 | 6 |
| --- | --- | --- | --- | --- | --- |
| strongly disagree | moderately disagree | slightly disagree | slightly agree | moderately agree | strongly agree |

3. I would try to solve this conflict even if my friend did not seem concerned.

| 1 | 2 | 3 | 4 | 5 | 6 |
| --- | --- | --- | --- | --- | --- |
| strongly disagree | moderately disagree | slightly disagree | slightly agree | moderately agree | strongly agree |

Citation:

Abeyta, A. A., Routledge, C., & Juhl, J. J. (2015). Looking back to move forward: Nostalgia as a psychological resource for promoting relationship goals and overcoming relationship challenges. *Journal of Personality and Social Psychology, 109,* 1029-1044.

**Participation in Social and Non-Social Research (Study 3 & 4)**

Thank you for completing this study. We have a number of forthcoming studies available for you to participate in. Many of these studies are paid. Next you will see a description of a couple studies. Indicate how interested you are in each study and whether or not you would be willing to participate.

**Study 1: Personality and Social Interaction**

This study investigates the process of meeting a new person. Research participants will be matched with and chat with another participant whom they do not know. The two participants will be given a number of topics to discuss. Some of these topics will delve into personal beliefs, opinions, and experiences. We are specifically interested in recruiting people with excellent social skills, who feel comfortable meeting new people and discussing various topics to participate in this study.

1. How interested would you be to participate in this study?

| 1 | 2 | 3 | 4 | 5 | 6 | 7 |
| --- | --- | --- | --- | --- | --- | --- |
| not interested |  |  |  |  |  | very interested |

1. Would you be interested in learning more about this study?

| 1 | 2 | 3 | 4 | 5 | 6 | 7 |
| --- | --- | --- | --- | --- | --- | --- |
| Definitely  no |  |  |  |  |  | definitely yes |

1. Would you like to participate in this study?

| 1 | 2 | 3 | 4 | 5 | 6 | 7 |
| --- | --- | --- | --- | --- | --- | --- |
| definitely  no |  |  |  |  |  | definitely yes |

**Study 2: Cognitive Problem Solving**

This study investigates how people solve problems, as well as ways to improve problem solving abilities. Participants will test out a new web application developed to assess peoples' ability to solve complex puzzles. The application is designed to give people feedback about their problem solving skills, as well as help them improve their problem solving skills. We are specifically interested in recruiting people who are good at and enjoy puzzles and games.

1. How interested would you be to participate in this study?

| 1 | 2 | 3 | 4 | 5 | 6 | 7 |
| --- | --- | --- | --- | --- | --- | --- |
| not interested |  |  |  |  |  | very interested |

1. Would you be interested in learning more about this study?

| 1 | 2 | 3 | 4 | 5 | 6 | 7 |
| --- | --- | --- | --- | --- | --- | --- |
| definitely  no |  |  |  |  |  | definitely yes |

1. Would you like to participate in this study?

| 1 | 2 | 3 | 4 | 5 | 6 | 7 |
| --- | --- | --- | --- | --- | --- | --- |
| definitely  no |  |  |  |  |  | definitely yes |

Citation:

Abeyta, A. A., Routledge, C., & Juhl, J. J. (2015). Looking back to move forward: Nostalgia as a psychological resource for promoting relationship goals and overcoming relationship challenges. *Journal of Personality and Social Psychology, 109,* 1029-1044.

**High Loneliness Experimental Condition (Study 4)**

The following survey is designed to assess your experiences with loneliness. Please indicate your agreement to each statement. After completing the survey, your responses will be tabulated and you will be given feedback on how you scored relative to your peers.

disagree slightly disagree slightly agree agree

1 2 3 4

1) I sometimes feel like I lack companionship

2) I sometimes feel that I am not close to people.

3) I sometimes feel that I do not have a lot in common with people around me.

4) I sometimes feel left out.

5) At times, I feel that nobody knows me well.

6) There are times when I feel isolated from others.

7) I sometimes feel like there is no one I can talk to.

8) I sometimes feel that people are around me but not with me.

9) Sometimes I feel that people really don’t understand me.

10) I sometimes feel like there is no one I can turn to.

Feedback:

Your loneliness score was [calculated sum]. This score is in the 67^th^ percentile of people in the United States. This score means that your level of loneliness is well above average.

**Low loneliness Condition (Study 4)**

The following survey is designed to assess your experiences with loneliness. Please indicate your agreement to each statement. After completing the survey, your responses will be tabulated and you will be given feedback on how you scored relative to your peers.

disagree slightly disagree slightly agree agree

1 2 3 4

1) I always feel like I lack companionship

2) I frequently feel that I am not close to people.

3) I always feel that I do not have a lot in common with people around me.

4) I always feel left out.

5) Very frequently, I feel that nobody knows me well.

6) Much of the time, I feel isolated from others.

7) I always feel like there is no one I can talk to.

8) I always feel that people are around me but not with me.

9) Much of the time, I feel that people really don’t understand me.

10) I always feel like there is no one I can turn to.

Feedback:

Your loneliness score was [calculated sum]. This score is in the 12^th^ percentile of people in the United States. This score means that your level of loneliness is very low.

**Nostalgia Condition (Study 4)**

**[Nostalgia condition]**

According to the Oxford Dictionary, 'nostalgia' is defined as a ‘wistful affection for the past.' Please bring to mind a nostalgic memory from your past that stands out in your mind as truly nostalgic. Specifically, reflect on your feelings of sentiment and longing for this memory.

Please write four keywords relevant to this nostalgic memory (i.e., words that sum up the gist of this memory).

__________________________

__________________________

__________________________

__________________________

Using the space provided below, for the next few minutes, we would now like you to write about the nostalgic memory. Describe this nostalgic memory and how it makes you feel warm and sentimental. Be as thorough as possible in describing how you are feeling.

**Reference:**

Wildschut, T., Sedikides, C., Arndt, J., & Routledge, C. (2006). Nostalgia: Content, triggers, functions. Journal of Personality and Social Psychology, 91, 975-993.

**Control Memory Condition (Study 4)**

Please bring to mind a memory. That is, think about an experience from your past. Specifically, reflect on your thoughts regarding this memory.

Please type four keywords relevant to this memory (i.e., words that sum up the gist of the experience).

__________________________

__________________________

__________________________

__________________________

Using the space provided below, for the next few minutes, we would now like you to write about the past event. Immerse yourself into this experience. Describe this past event and what it makes you think about and feel. Be as thorough as possible in describing what you are thinking.
